# Livestock Genomics for Developing Countries – African Examples in Practice

**DOI:** 10.3389/fgene.2019.00297

**Published:** 2019-04-24

**Authors:** Karen Marshall, John P. Gibson, Okeyo Mwai, Joram M. Mwacharo, Aynalem Haile, Tesfaye Getachew, Raphael Mrode, Stephen J. Kemp

**Affiliations:** ^1^Livestock Genetics Program, International Livestock Research Institute, Nairobi, Kenya; ^2^Centre for Tropical Livestock Genetics and Health, Nairobi, Kenya; ^3^School of Environmental and Rural Science, University of New England, Armidale, NSW, Australia; ^4^Small Ruminant Breeding and Genomics Group, International Center for Agricultural Research in the Dry Areas, Addis Ababa, Ethiopia; ^5^Scotland’s Rural College, Edinburgh, United Kingdom

**Keywords:** livestock, Africa, genomics, smallhold, SNP, breeding program, genetic improvement strategy

## Abstract

African livestock breeds are numerous and diverse, and typically well adapted to the harsh environment conditions under which they perform. They have been used over centuries to provide livelihoods as well as food and nutritional security. However, African livestock systems are dynamic, with many small- and medium-scale systems transforming, to varying degrees, to become more profitable. In these systems the women and men livestock keepers are often seeking new livestock breeds or genotypes – typically those that increase household income through having enhanced productivity in comparison to traditional breeds while maintaining adaptedness. In recent years genomic approaches have started to be utilized in the identification and development of such breeds, and in this article we describe a number of examples to this end from sub-Saharan Africa. These comprise case studies on: (a) dairy cattle in Kenya and Senegal, as well as sheep in Ethiopia, where genomic approaches aided the identification of the most appropriate breed-type for the local productions systems; (b) a cross-breeding program for dairy cattle in East Africa incorporating genomic selection as well as other applications of genomics; (c) ongoing work toward creating a new cattle breed for East Africa that is both productive and resistant to trypanosomiasis; and (d) the use of African cattle as resource populations to identify genomic variants of economic or ecological significance, including a specific case where the discovery data was from a community based breeding program for small ruminants in Ethiopia. Lessons learnt from the various case studies are highlighted, and the concluding section of the paper gives recommendations for African livestock systems to increasingly capitalize on genomic technologies.

## Introduction

In developing countries, the livestock sector plays a key role in the provision of livelihoods as well as food and nutrition security. The majority of livestock are kept by the rural poor, where they serve multiple functions. These include: savings and insurance, food security (meat and milk), income, livelihood diversification and thus risk reduction (such as in mixed crop-livestock systems), inputs to crop production (draft power, manure as fertilizer), transportation, various uses of hides and skin (such as for housing), allowing households to benefit from common-property resources (such ascommunal grazing areas), and fulfilling social obligations (such as being used in special ceremonies or for dowry), amongst other (Herrero et al., 2013; Marshall, 2014; Marshall et al., 2014; ILRI, 2019). The livestock sector also benefits other actors in the associated value chains, such as input providers, traders, processors and retailers, through the provision of employment and income. Critically, animal source foods – consumed in even small amounts - play a key role toward food and nutritional security of the poor, as they provide quality protein and micronutrients essential for normal development and good health (Grace et al., 2018; Smith et al., 2013).

The demand for animal source foods is rapidly increasing in developing countries: for example, in low income countries the demand in 2030 for beef, milk, poultry and eggs is predicted to be a 124, 136, 301, and 208% increase over that in 2000, respectively (FAO, 2011). This demand increase has been largely attributed to population growth, income growth and increasing urbanization (Delgado, 2005; Thornton, 2010). To ensure this demand is met, large increases in livestock production within developing countries will be required (Delgado et al., 2001; Steinfeld et al., 2006; Thornton, 2010). Achieving this in a sustainable manner is expected to be challenging, with a key component of this recognized to be increasing livestock productivity (output per unit of input).

Increasing livestock productivity in developing countries generally requires simultaneous interventions in the areas of animal feed, health and genetics. In many livestock development programs these interventions take the form of capacity building of the livestock keepers and other value chain actors, ensuring the availability and accessibility of inputs, provision of new technologies or customization of existing technologies, support to private and/or public sector involvement, and advocacy for supportive policies. The provision of incentives for increased productivity can also be important, such as in some small-hold and pastoral sectors where livestock are primarily kept for savings and insurance purposes, so maintaining a livestock asset base is more important to the household than improving livestock productivity. Such incentives could be provided by, for example, increasing livestock income through facilitating access to strong and stable markets, or ensuring that intra-household benefit from the livestock enterprise is equitable. In addition, attention to other issues which can be affected through increased livestock productivity, such as equality, food safety and environmental sustainability, are also commonly part of livestock development programs. As livestock systems within developing countries are both diverse and dynamic, intervention packages typically need to be customized for each livestock sector.

To date, the majority of African livestock systems have not benefited from livestock technologies to the extent that developed countries have, including in relation to genetic improvement strategies (Marshall, 2014). Currently, there are few examples of sustainable breeding programs and the use of reproductive technologies, such as artificial insemination, is limited to specific livestock sectors. Contributing factors to this include: the lack of public and private sector investment; lacking or weak supportive policies and institutional arrangements; the heterogeneity of livestock systems, farm-scales, management practices, and needs and preferences of livestock keepers; poor infrastructure; and limited capacity in the field of animal breeding and reproduction, amongst other (Kosgey and Okeyo, 2007; Rege et al., 2011; Marshall, 2014). The potential of genetic improvement to increase livestock productivity is, however, increasingly being recognized by decision makers, with many African countries now explicitly including genetic improvement within their national livestock development plans.

The types of structured genetic improvement programs being implemented in Africa vary by system. These include: breed-substitution with other African breeds, breeds from other tropical countries such as India and Brazil, as well as breeds from elsewhere; cross-breeding, most commonly where a highly adapted but lowly productive indigenous breed is crossed with a poorly adapted but highly productive exotic breed; and less commonly within-breed improvement (FAO, 2015). Increasingly, explicit attention is being paid to the development of working models to ensure sustainability of these programs, as it has been well demonstrated that the models implemented in developed countries cannot be directly applied. The application of genomics – ranging from the determination of breed composition of animals in the absence of pedigree data for *in situ* comparison studies, or for the application of genomic selection in breed improvement programs – is just beginning to emerge, often overcoming a constraint that would otherwise exist, such as lack of recorded pedigree.

In this article we describe several examples of the use of genomics in sub-Saharan African livestock systems, draw lessons learnt from these, and giving recommendations for African livestock systems to increasingly capitalize on genomic technologies. The paper proceeds as follows. The subsequent section ‘case studies’ describes the case studies grouped by application, namely the use of genomic information to: (1) to identify the most appropriate breed or cross-breed type for different livestock production systems; (2) to enable or enhance breeding programs; (3) create new breed-types; and (4) discover genetic variants of economic and ecological significance. A discussion follows, first addressing current developments on livestock genomics in Africa, drawing on the case studies, and secondly describing the future outlook for livestock genomics in Africa.

## Case Studies

### Use of Genomic Approaches to Aid Identification of the Most Appropriate Breed or Cross-Breed for Different Livestock Production Systems

Identification of the most appropriate livestock breed or cross-breed type in a particular livestock production system is typically the starting point of a genetic improvement strategy. In African livestock systems that are undergoing intensification this is particularly relevant (Marshall, 2014). To-date there are few studies to this end due to lack of investment in this area plus, in the case of cross-breeds, the inability to assign breed-type to animals in the field which is necessary for *in situ* comparisons (see Marshall, 2014 for a review). The latter stems from the lack of pedigree information in most African livestock systems and the near impossibility of assigning breed-type based on phenotype, particularly in systems where unstructured cross-breeding is prevalent. The use of genomic approaches to assign breed composition to individual animals can overcome this constraint (Kuehn et al., 2011; Ojango et al., 2014). Here we discuss examples for dairy cattle systems in Senegal and Kenya, and sheep systems in Ethiopia.

#### Kenya Dairy Cattle

In Kenya the large majority of milk is produced by smallholder farmer who typically milk 1–5 cows. Smallholders mostly keep crosses between indigenous cattle and exotic dairy breeds such as Holstein, Friesian, Ayrshire, and Jersey. There is no systematic breeding of crossbred cattle and farmers rarely keep pedigree or performance records. Most mating events involve local crossbred or indigenous bulls, where the crossbred bulls are of unknown breed composition. Farmer production environments vary greatly and this translates into a wide range of production output per cow, from less than 1,000 l milk per annum to more than 5,000 l, with the large majority likely in the range 1,000 to 3,000 l milk. There is no information about which breed composition works best for different production environments, other than the general observation that high grade exotics (cows with a very high proportion of exotic dairy breed composition) can do well in very good environments. The likelihood is that the intermediate grades do better in poorer production environments but given the lack of evidence most advice provided to farmers is that they should upgrade to higher grade exotic animals by using AI.

The Dairy Genetics East Africa project set out to determine what grade of crossbred (i.e., what percentage of exotic dairy breed composition in a crossbred cow) worked best for different production environments. The project worked with farmers to collect performance data, including on milk yields, reproduction events, and disease incidence, for 18–24 months. Further the recorded animals were genotyped using the Illumina bovine high density (HD, 780 k) single nucleotide polymorphism (SNP) assay with the HD SNP data used to perform admixture analyses, using the ADMIXTURE software (Alexander and Novembre, 2009), to generate an estimate of ancestral breed composition of each animal. This allowed, for the first time, accurate information on breed composition to be combined with *in situ* performance data to determine what breed composition worked best in different smallholder environments. By comparing farmer and enumerator (field staff) assessments of breed composition, based mostly on phenotypic appearance and farmer recollections on cows origins, with the admixture determinations of actual breed composition it was confirmed that phenotype-based assessments were very poor predictors of actual breed composition (*R*^2^ = 0.16). The results showed that intermediate to low grade (<50% exotic breed ancestry) cows performed best in the majority of the smallholder farms, while animals with higher grades (>50%) only performed better than lower grades in the best environments (those supporting >1800 l/cow/annum: Ojango et al., 2014).

A surprising result of this study was that average production levels (approximately 1,500 l/cow/annum) of the cows in the study, which were randomly sampled based on location in order to achieve a representative sample, was much lower than the 3,000–5,000 l/cow/annum typically assumed in most development projects and extension programs. The highest yielding cow in the study only achieved around 2,400 l/cow/annum. The result meant that it could not be inferred at what level would high grade exotic crossbreds or purebred exotics become the best performing breed type. The results also mean that most development and extension programs are making unrealistic assumptions about smallholder production environments and are likely, therefore, to be offering suboptimal or unrealistic interventions and advice. This is mentioned here because studies such as Dairy Genetics East Africa have multiple objectives in studying what are highly complex systems. As such, genomics is a powerful tool that assists better understanding of system function that should be incorporated into multidisciplinary studies rather than used to tackle isolated (genetic) issues. In the case of Dairy Genetics East Africa the results that were enabled by the use of genomic testing provided much of the baseline information that demonstrated the value and feasibility of establishing long-term genetic improvement programs, beyond the provision of the most appropriate breed cross, which led to the establishment of the Africa Dairy Genetic Gains (ADGG) program, which appears as another case study later in this review.

#### Senegal Dairy Cattle

In Senegal, dairy production is mainly from cattle kept in low input systems, with domestic production unable to meet national demand. To increase national dairy production the Senegalese government has implemented a number of initiatives, including the introduction of exotic high-yielding dairy breeds through public artificial insemination campaigns. However, at the time of these campaigns there was no evidence base for Senegalese cattle keepers and other stakeholders to make informed decisions on which dairy breed or cross-breed to use. This knowledge gap was addressed by a project termed “Senegal Dairy Genetics” which aimed to identify the most-appropriate dairy cattle breed/cross-breed for Senegalese production systems.

Project data was obtained by monitoring 220 rural or peri-urban dairy cattle keeping households, with collectively more than 3,200 cattle, over an almost 2 year period. Data collected included that on animal performance, economics of the household dairy enterprise, social issues including on gender, and dairy cattle feed and milk safety, amongst other. The aim was to collect a range of data such that different household dairy systems (defined as a combination of breed-type kept and level of animal management) could be compared from multiple perspectives including milk-yields, household profit and cost:benefit ratio, and food safety (Marshall et al., 2016b, 2017; Salmon G. et al., 2018).

The main breeds and cross-breeds of cattle kept by the project households comprised pure indigenous Zebu, indigenous Zebu crossed with Guzerat, indigenous Zebu crossed with *Bos taurus* breeds (such as Montbéliard and Jersey) and pure (or almost pure) Bos Taurus breeds. With the exception of the indigenous Zebu, the breed-type of individual animals was not able to be determined based on phenotype, and none of the cattle keepers kept pedigree records. Thus breed composition of a subset of the study animals, those with the most informative records, was determined using a genomic approach. Specifically, genotyping was performed using the Bovine 50 K SNP assay and admixture analysis performed. using the Bayesian Analysis of Population Structure software (Corander et al., 2008). Animals were each assigned proportions of ancient Zebu, recent Zebu, ancient Taurine and recent Taurine, and from here assigned to breed-groups: see Tebug et al. (2016) for more details. In comparing breed-group assignment from the genomic analysis to that based on farmer-stated breed-type there was only a match in 32% of the cases.

Following breed-composition assignment of the study animals, trade-off analysis proceeded for the various household dairy systems (Marshall et al., 2017; Salmon G. et al., 2018). Notably it was found that cross-bred indigenous zebu by *Bos taurus* dairy cattle kept under better management produced up to 7.5-fold higher milk-yields, 8-fold higher household profit, and 3-fold lower greenhouse gas emission intensity, per cow per annum, in comparison to indigenous Zebu kept under poorer management, for a typical herd size of eight animals (Marshall et al., 2016b; Salmon G.R. et al., 2018). Trade-offs to this were that the cross-bred cattle consumed more supplementary feed, some of which was aflatoxin contaminated which can result in milk unfit for human consumption (Marshall et al., 2016a), and that as the household dairy enterprises commercialized (associated with the keeping of cross-bred dairy cattle) there was a partial shift in the control of income from milk sale from women to men (Walugembe et al., 2016).

Results of the study were shared with decision makers on dairy in Senegal, including women and men dairy cattle keepers, other value-chain actors, and policy makers, for better-informed decision making. Discussions with these stakeholders are currently underway to implement a livestock development program aimed at increasing the availability and accessibility of cross-bred animals, whilst addressing the known trade-offs. Similar to the Kenya Dairy case study above, this highlights the use of genomics in multi-objective studies.

#### Ethiopia Sheep

Crossbreeding local sheep with usually much bigger exotic breeds has been common practice in many countries of Africa over the last five decades (Getachew et al., 2016). Generally, performances and adaptability of crossbreds greatly varied by location, management and exotic inheritance level (Getachew et al., 2013, 2016). In Ethiopia, the common approach is to upgrade local breeds by repeatedly back crossing to high level exotic sires, mainly of the Awassi and Dorper breeds. However, it is difficult for farmers and other stakeholders to make informed decisions on which level of cross (in terms of local versus exotic contribution) to aim for, due to lack of evidence to this end. This was addressed in the highlands of Ethiopia by a project aimed at associating cross-breed type with performance, as described here.

Study data was obtained from an on-going crossbreeding program being implemented in the Amhara region of the Ethiopian highlands (Gizaw and Getachew, 2009). This crossbreeding program has been ongoing since 1998 and involves crossing of the local Menz and Wollo breeds to the exotic Awassi breed, with a wide range of crossbreeds produced. Phenotype data collection on lamb growth and ewe reproductive was routine in the breeding programs. However, the breed composition of the animals was unknown as pedigree had not been recorded (due the practice of communal grazing).

Genomics helped to estimate breed proportion in the absence of pedigree recording with, specifically, breed-composition assigned to individual animals using a reduced set of ancestry informative markers (AIM). The AIM were selected from Ovine SNP50K data from the Menz, Wollo, and Awassi breeds. A total of 74 SNP that showed large differentiation between the local Menz and Wollo breeds to the Awassi breed were selected based on their *F*_ST_ values. These accurately (*r* = 0.98) identified the breed proportion of reference samples (which comprised pure Awassi, 75% Awassi and 50% Awassi), as did sub-sets of 65, 55, and 45 SNPs selected on high or low *F*_ST_ values (with correlations of 0.9996 to 0.969 between breed estimates from these subsets and the 74 SNP; Getachew et al., 2017). The small number of AIM required is consistent with studies in human populations (Halder et al., 2008).

More than 700 animals, presumed to have a wide range of breed compositions, were genotyped using selected AIMs. Breed proportion of individual animals was then determined and related to ewe productivity expressed as 8 months lamb weight per year (considered a useful combined trait comprising growth, reproduction and lamb survival). The most productive breed compositions were then identified as 37.5–50% Awassi in the first study site, and12.5–25% Awassi in the second study site where ewes produced (on average) 26.5 and 19.5 kg lamb, respectively, at 8 months (Getachew et al., 2017). Findings of this project were shared with various local research centers with recommendations from the project adopted. Accordingly, crossbreeding in the first study site is moving toward synthetic breed development, whilst cross-breeding in the second study site was discontinued due to perceived unfavorable economic benefits (i.e., high cost:benefit ratio).

The AIM is considered a great opportunity to estimate the level of admixture (breed proportion) in a cost-effective way. Currently, the cost per SNP is in the range of about €0.04–0.15 for low density panels, highly dependent on the method and number of samples to be genotyped at a time. It is of note that information on ram breed composition (based on visual assessment and in some cases partial pedigree) is currently used in ram marketing, and that many farmers within the study site showed interest to pay for breed composition information. If an affordable tool (based on a low-cost SNP chip) was available for this purpose, ram sellers would be better placed to take advantage of the market opportunity for rams of known breed-type.

### Use of Genomic Approaches to Enable or Enhance Breeding Programs

In intensive livestock systems, genomic data enhances existing genetic improvement programs by increasing the accuracy of estimates of relationships among animals, and hence increasing accuracy of estimated breeding value (EBV), and in some cases also revealing functional variants which can be selected for directly using genotype data. The big immediate advantage of genomic data in Africa is to enable rapid implementation of genetic improvement where pedigree information is lacking, which is commonly the case. In such cases genomic data can be used to build a genetic relationship matrix among animals in a new recording program, so that EBV can be generated almost immediately. Where genetic relationships are based on pedigree recording, EBV cannot be generated until the next generation of animals have been born and recorded. Similarly, once phenotype and pedigree recording programs are in place, genomic data allows rapid expansion of recording to include animals with no previous pedigree information. Where genetic improvement systems are well established in Africa, genomic data potentially offers the same technical benefits as in intensive livestock systems. An additional advantage in crossbred populations is that genomic data can be used to accurately determine breed composition of individual animals and this information can be used to increase the accuracy of genetic evaluations and breed effects, in addition to being used directly to select animals of desired breed composition. In the case of pure breed populations, estimates of breed composition can also be used to ensure the purity of the breed. The case study below is an excellent example highlighting how genomics has facilitated a breeding program in an African livestock system.

#### East Africa Dairy Cattle

In the smallholder, crossbred dairy system that dominate sub-Saharan milk production, the lack of performance and pedigree recording means that there are no conventional genetic evaluation systems for these systems (Kosgey and Okeyo, 2007). In addition, indiscriminate crossbreeding has been undertaken, with no clear goal in mind, thus leading to populations of highly varied breed composition and no information about the breed composition of individual animals. Two initiatives in East Africa, the Dairy Genetics for East African program (described above) and the African Dairy Genetic Gains program funded by the Bill and Melinda Gates Foundation have explored routes to establishing relevant and sustainable genetic improvement programs by combining genotype information with establishment of effective performance and pedigree recording.

Genotype data from high-density SNP assays can offer quick wins in smallholder systems. SNP data can be used to assign parentage where pedigree data is not available. Knowledge of breed composition of bulls allows farmers to use bulls of the breed composition they desire, and having cows with known breed composition allows farmers to determine what breed composition of bulls is required to produce progeny of the desired breed type. Further, knowing the breed composition of cows and bulls allows purchasers of animals to obtain animals with the breed composition required for their production environment. As illustrated in the case studies described above, the same approach can be used to determine breed composition in studies that determine the optimum breed composition for different production environments, thereby informing farmers what breed composition of cow or bull they should be aiming to purchase or to produce through breeding.

Commercially available SNP assays are currently too expensive to allow their routine commercial use in parentage assignment and determination of breed composition in East African dairy systems. However, using the Dairy Genetics East Africa high-density genotype data on 2940 crossbred cattle in East Africa (Kenya, Uganda, Ethiopia, and Tanzania), Strucken et al. (2017) developed reduced SNP panels consisting of 200–400 SNPs each; one set of panels for the accurate determination of breed composition and the other set for accurate parentage verification. These assays will soon be tested in the field to determine the feasibility of delivery on a large scale at a price farmers and others are willing to pay, with a target of $10–$20 for laboratory costs. If smartly and widely used, these tools will enable almost immediate genetic improvement through targeting of the best genotypes to different production environments, which in turn will allow the formation of synthetic dairy breeds in which long-term genetic improvement can be practiced.

The availability of genotypic data has enabled the estimation of genetic parameters and the estimation of genomic breeding values for milk yield in these populations using the G matrix obtained from SNP genotype data (Brown et al., 2016; Mrode et al., 2018). Using milk test day records on 1034 cows and genotypes from the Dairy Genetics East Africa project, Brown et al. (2016) applied genomic best linear unbiased prediction (GBLUP) and Bayes C models to examine the accuracy of genomic predictions for cows of different breed composition. The study reported accuracies of genomic prediction varying from 0.30 to 0.40. Using the same dataset, Mrode et al. (2018) examined models with dominance effect and a multi-trait approach fitting breed proportion as separate traits. Although the dominance effects were essentially zero, possibly due to the small size of the dataset, the multi-trait approach resulted in a slight improvement in the predictive ability of the model, although not in accuracy of prediction, compared to the results of Brown et al. (2016). While the accuracies reported in these studies in East Africa are lower than estimates from developed countries (Wiggans et al., 2017), they are very promising given the limited data sets and the fact that there is no existing breeding program with which these genomic EBV (gEBV) for crossbred performance have to compete. The results highlight the need for more data and the consequent advantage of pooling data across countries in future (Mrode et al., 2018). The Bill and Melinda Gates Foundation funded African Dairy Genetic Gains (ADGG) project is generating more data across two countries and would offer more opportunity to further examine the application of GS in small holder system (Mrode et al., 2018). The intention is to initiate routine genomic evaluations, and selection and recruitment of young bulls for use in the National Artificial Insemination Centers (NAIC) in Tanzania, Ethiopia, and Kenya. In addition, genome wide association studies (GWAS) are planned to determine whether genes or genetic regions controlling production and reproduction traits can be identified that can be used to further enhance genetic improvement in these populations.

To improve the cost-effectiveness of applying genomic selection in East Africa, the feasibility of developing a reduced (i.e., cheaper) chip for genomic prediction was examined using the 3,513 animals with high density genotypes in the Dairy Genetics East Africa data (Aliloo et al., 2018). Various methods were examined for selecting panels with reduced number of SNP for imputation and genomic prediction within the crossbred populations. It was found that a specially developed (co)variance method that accounted for the covariance between adjacent SNPs and the minor allele frequency of SNPs, out-performed other approaches such as using the minimum minor allele frequency or random SNP selection. High accuracies of imputation of about 0.80 and 0.94 were observed when imputing from optimized 7 K and 40 K panels to HD. The use of these LD data imputed to HD was accompanied by a high accuracy of genomic prediction of about 0.98 compared to use of unimputed HD data. The highest imputation accuracy were obtained with a reference population consisting of a mixture of crossbred and ancestral purebred animals. As the cost of existing commercial genotyping assays continues to fall, the value of having smaller customized assays is reducing, and, with current technologies, they may well become more expensive than commercial assays that are used globally because of their more limited use. Innovative applications of genomic technology or tools for breed composition and parentage determination, and genomic prediction, if accompanied by sound business models for their delivery hold great potential for impact in Africa.

### Use of Genomic Approaches in the Creation of New Breed-Types

The most appropriate breed-type for African livestock systems are typically considered those which are both productive and adapted/resilient. Genomics and its associated technologies/techniques (transgenesis, cloning, gene/genome editing etc.) offer opportunities for creating such breed-types. The below case study is one example of this.

#### Trypanosome Resistant Cattle

Animal trypanosomias is caused by a group of extracellular protozoan parasites and transmitted by the tsetse fly (*Glossina* spp.) is a major constraint to livestock production across much of the African continent with massive economic consequences (Kristjanson et al., 1999; Shaw et al., 2014). Attempts to develop vaccines against this pathogen have largely failed due to its ability to rapidly change its highly antigenic surface glycoprotein (La Greca and Magez, 2011). The alternative prevention measure, tsetse vector control has proved expensive and difficult to sustain with adverse environmental consequences (Tirados et al., 2015). However, some African *Bos taurus* cattle breeds, such as N’dama, are tolerant of infection with trypanosomes, remaining healthy and productive and without the anemia that is characteristic of infection in susceptible breeds. This phenomenon has been termed trypanotolerance. Importantly trypanotolerant animals continue to harbor parasites and can succumb to pathology under physiological stress (Murray et al., 1984).

Because of the difficulty in conventional control methods, there has been significant research into a genetic approach to enabling livestock production under trypanosome challenge. In a series of studies, quantitative trait loci influencing response to trypanosome challenge were mapped in a mouse model (Kemp et al., 1996) and in N’dama cattle (Hanotte et al., 2003). Eventually, a combination of linkage mapping, expression analysis, candidate gene sequencing, population analysis and *in vitro* studies allowed candidate genes and variants to be identified with some confidence (Noyes et al., 2011). However, no genes of large effect were identified and the mechanism of tolerance remains unclear.

An alternative genetic-based approach is currently under investigation that attempts to exploit the resistance to infection with some trypanosome species shown by most primates. Resistance in primates is mediated by subset of high-density lipoproteins (HDLs) called trypanosome lytic factors (TLFs) which kill many trypanosome species (Thomson et al., 2009). The active component of TLF has been shown to be apolipoprotein (apoL-1) which, following endocytosis by the trypanosome, is activated within the acidic lysosome to form membrane pores, resulting in parasite swelling and lysis (Molina-Portela Mdel et al., 2005; Thomson and Finkelstein, 2015). Primate TLF has been shown to kill the cattle-infective trypanosome, *Trypanosoma congolense* as well as the human-infective trypanosomes, *T. brucei rhodesiense*. Furthermore susceptible mice have been shown to become fully resistant to infection with these trypanosomes following transfection with primate-derived APOL1 (Thomson et al., 2009). There is thus good reason to believe that transgenic cattle could be constructed, which are fully resistant to trypanosomes. This could potentially allow *Bos indicus* cattle breeds that are well adapted to the African environment, except for susceptibility to trypanosomes, to become sustainably resistant without the use of toxic drugs or environmentally damaging insecticides and research to explore this possibility in East Africa is currently underway (Lukeš and Raper, 2010; Yu et al., 2016).

### African Indigenous Livestock as Resource Populations for Discovery of Genetic Variants of Economic and Ecological Significance

African livestock populations are rich resources for discovery of genetic variants, and many efforts are underway to this end. The first case study below describes a breeding program for small ruminants (sheep and goats) which, whilst currently not using genomics as part of the breeding program itself, is using the breeding program data for genetic variant discovery purposes. Following this a second ‘case study’ illustrates other efforts toward genetic variant discovery: unlike the other cases described here which are specific initiatives/projects, this draws on numerous studies to showcase the various types of activities occurring in this space.

#### Ethiopia Small Ruminants

In small ruminants, centralized breeding schemes, entirely managed and controlled by governments – with minimal, if any, participation by farmers – were developed and implemented in many developing countries. Such programs have generally failed to sustainably provide the desired genetic improvements to smallholder livestock keepers. Community-based breeding programs have been suggested as an alternative and are being implemented in a few pilot countries. Programs that adopt this strategy consider the farmers’ needs, views, decisions, and active participation, from inception through to implementation, and their success is based upon proper consideration of farmers’ breeding objectives, infrastructure, participation, and ownership (Sölkner et al., 1998; Mueller et al., 2015). Community-based breeding programs in Ethiopia started in 2009 and currently cover 3,200 households keeping more than 48,000 sheep and goats. The goal of the program is to improve the productivity and income of these small-scale resource-poor sheep and goat producers by providing access to improved animals that respond to improved feeding and management, facilitating the targeting of specific market opportunities (Haile et al., 2011, 2018).

A study using selected animals recorded as part of the community-based breeding program was performed toward identifying genes for prolificacy. Here 84 sheep giving either single, twin, triplet, quadruplet etc. birth types were used in a signatures of selection study to identify candidate genes for prolificacy. Animals giving single births (20) were taken as controls while those giving multiple birth (64) formed the cases. *F*_ST_ analysis revealed two candidate regions, one on chromosome 5 and the other on the X. The latter was the most significant. hapFLK identified the region on the X only. The candidate region on chromosome 5 was adjacent to *GDF9* and the region on the X spanned the *BMP15* (*GDF9B*) gene. These two genes are expressed in oocytes and have been shown to be essential for ovulation rate, normal follicular growth and maturation of preovulatory follicles (McNatty et al., 2004). From examination of inherited patterns of ovulation rates in other sheep, point mutations have been identified in both genes. Animals heterozygous for any of these mutations have higher ovulation rates (that is, +0.8–3) than wild-type contemporaries, whereas those homozygous for each of the mutations are sterile with ovarian follicular development disrupted during the preantral growth stages. The genes are being sequenced to identify the point mutations and once confirmed, strategies to introgress the alleles conferring prolificacy into other, non-prolific, populations would be designed.

#### Other Initiatives on Genetic Variant Discovery

Post domestication, livestock genomes have continuously been modified through selective breeding for economically or otherwise important traits, and natural selection for adaptation to local agro-environments. Africa has diverse agro-environments and a predominantly tropical environment that is characterized by harsh and extreme climatic conditions, seasonal feed and water scarcity, heat stress, high solar radiation, widespread pathogens, parasitic infections and disease epidemics. These present the main evolutionary forces shaping Africa’s livestock genomes. Accordingly, African livestock display unique adaptive traits including enhanced disease resistance, superior innate immunity and greater ability to thrive, produce and reproduce in unfavorable environments. Some of the adaptive traits in African livestock, such as resistance to gastro-intestinal parasites in small ruminants, are of global significance.

There are numerous African livestock populations already identified as of interest for gene-discovery studies. These include, as examples: breeds that are highly resistant/tolerant to gastro-nematodes, such as the Red Maasai sheep and Small East African Goats of East Africa, West African Dwarf sheep and Goat (Preston and Allonby, 1978; Baker et al., 1999, 2003; Goossens et al., 1999; Behnke et al., 2006); breeds from West Africa that exhibit strong trypanotolerance, such as the N’dama, Somba, Baoulé, Lagune and Muturu cattle, and West African Dwarf sheep and goat (Agyemang, 2005; Geerts et al., 2009; Berthier et al., 2016); cattle breeds that produce “robust” milk yields in harsh conditions, such as the Butana and Kenana of Sudan (Peters et al., 2005; Salim et al., 2014); Zebu cattle that demonstrate innate ability to regulate body temperature under heat stress by maintaining lower metabolic rates and rectal temperatures, lower respiratory rates and lower water requirements (Gaughan et al., 1999; Hansen, 2004); and breeds that are highly prolific, such as the sheep breeds of Djallonké from West Africa (Tuah and Baah, 1985), Bonga, Horro, and Arsi-bale from Ethiopia (Rekik et al., 2015), D’Man from Morocco (Aherrahrou et al., 2015), and Barbarine from Tunisia (Lassoued et al., 2017).

There are an increasing number of examples of African livestock populations being used in studies aimed at identifying the genes or gene-pathways and genomic variants underpinning economically or ecologically important traits. These include a number of studies that have detected putative signatures of selection for a variety of traits including feeding/drinking behavior, heat tolerance/thermoregulation, tick resistance, milk production under harsh environments, immune response, meat quality, and reproductive performance, amongst others (Makina et al., 2015; Mwacharo et al., 2017; Taye et al., 2017; Bahbahani et al., 2018). There are additionally some reports of GWAS, such as for tick and gastrointestinal parasite resistance (Benavides et al., 2015; Mapholi et al., 2016), though these are rarer due to lack of datasets with both phenotypes and genotypes recorded on sufficient animals. Some genetic mapping studies targeting QTL identification, such as for resistance to gastro-intestinal nematodes and trypanotolerance (Hanotte et al., 2003; Marshall et al., 2013), have also been reported. In cases candidate genes have also been identified within the genomic regions of interest, for instance genes likely associated with trypanotolerance (Berthier et al., 2016). Should this work be extended to the identification of refined genomic regions and/or validated functional mutations and variants, there is potential for it to be fed into genetic improvement strategies, either via breeding programs incorporating the use of genomic/genetic data or through the creation of new breeds via either introgression or genome modification approaches.

An exciting possibility in crossbred dairy cattle populations such as those in the Dairy Genetics East Africa project is that as data increases it will be possible to undertake GWAS to identify genetic regions, and potentially the genes, controlling genetic variation in milk production and adaptation traits. The differences between exotic dairy breeds and indigenous breeds in their genetic potential for milk production and in adaptation traits are larger than for any other crosses of livestock. For example the genetic potential for milk production of Holstein cattle is about 10-fold higher than that of indigenous breeds such as the Small East African Zebu. GWAS may be able to identify the genetic regions that control these massive genetic differences between breeds. However, GWAS in crossbred cattle presents some challenges. In a purebred population GWAS is based on population-wide linkage disequilibrium (LD) between SNP and functional genetic variants. In a crossbred population there are at least three forms of LD: the LD coming from within the indigenous ancestors; the LD from within the exotic ancestors; the between population LD, which is the LD generated within the crossbreds due to segregation of loci that were fixed for opposite alleles in the exotic vs. indigenous ancestors. In practice the problem is even more complicated because most LD is not conserved between exotic or between indigenous breeds, so each of the various ancestral dairy breeds and indigenous breeds injects different amounts and phases of LD, reducing further the average LD observed in crossbred populations. It is not yet clear whether existing SNP assays provide sufficient information to track inheritance of segments of the genome back to their diverse origins with sufficient accuracy to undertake GWAS that separates the different forms of LD in the population. Assuming that it will prove possible, the between population LD is potentially of greatest interest given the very large genetic differences between ancestral breeds and the potential to apply gene-based selection for suitable combinations of productivity and adaptation traits. Very low density, and hence potentially cheap, assays of a few hundred SNP might be developed and applied to widely test animals and select those with optimum combinations of productivity and production variants, even if genotyping with commercial assays proves too expensive for routine use in genetic improvement in these systems.

An additional body of work has focused on characterizing genetic diversity, population structure and relationships in African livestock (Hanotte, 2002; Missohou et al., 2006; Muigai and Hanotte, 2013; Decker et al., 2014). Such studies are useful in understanding their evolutionary history as well as identifying appropriate populations for the identification of genomic variants.

## Discussion

### Current Developments

The case studies presented show-case a number of livestock genomic technologies currently being applied or piloted in livestock systems of sub-Saharan Africa. These included those aimed at identifying the most appropriate breed-type for particular production systems/environments, a breeding program incorporating genomic selection as well as parentage and breed composition determination, an initiative aimed at creating a new breed-type, and efforts toward discovery of genetic variants. Other examples outside of those presented here also exist within sub-Saharan Africa, with, in particular, major efforts in South Africa to incorporate genomic selection into established breeding programs for a number of species (van Marle-Köster et al., 2013; Cloete et al., 2014; Westhuizen and van der Marle-Köster, 2014; Mohlatlole et al., 2015; Prescilla et al., 2015). These examples are all fairly recent, mostly emerging within the last 5 years, and highlight the developing use of genomics in African livestock systems.

It is of note that the differences in livestock production systems, and type of genetic improvement strategy used within them, between developed countries and Africa (as discussed in the introductory section of the paper) have led to different emphasis on how genomics is currently being applied. In developed countries the most suitable animal genetic resources for a particular production systems is usually well established, whereas in many African production systems, and particularly those undergoing change such as through intensification, there is generally little evidence to make such recommendations (Marshall, 2014). The use of genomic data to determine the breed-type of admixed animals’ monitored *in situ* (i.e., kept by farmers) has been transformational to this end, as it has removed the high error of assigning breed-type of admixed animals based on observation (phenotype) or farmer recall. Genomic selection is now common-place is many developed country livestock breeding programs, whereas in Africa it is in its infancy. This principally stems from the lack of breeding programs into which genomic selection can be implemented, with some notable exceptions including the African Diary Genetics Gains initiative described here and various breeding program in South Africa, many of which are working on developing sufficiently sized reference populations to incorporate genomic selection (van Marle-Köster et al., 2013; Cloete et al., 2014; Westhuizen and van der Marle-Köster, 2014; Mohlatlole et al., 2015; Prescilla et al., 2015). In the case of African Dairy Genetics Gains, the use of genomic information has overcome the constraint of lack of pedigree data, enabling a breeding program where it would have previously been difficult, if not impossible. African Dairy Genetics Gain is also piloting the use of genomic technologies for parentage verification as well as breed composition determination (particularly for cross-bred bulls) with a view to potential commercialization, the success of which will likely depend on whether there is sufficient market demand, in-turn linked to whether the technologies can be sold at a price affordable to African livestock keepers.

Using genomics to aid the development of new breed-types for African livestock systems has received limited attention to date. Given the high interest in developing new breeds that have the adaptation and resilience of indigenous breeds combined with the productivity of exotic breeds, and the difficulty in many systems of maintaining a structured cross-breeding program, the cost:benefit of using genomic approaches to create an adapted and productive synthetic breed, in comparison to traditional approaches, is worth exploring in the African context. On the creation of new breed-types via transgenic or gene-edited approaches, few validated genes of interest currently exist. One notable exception to this is the gene conferring resistance to trypanosomiasis as described in the case study presented here. Other variants of potential interest are those underpinning the slick hair phenotype, given this phenotypes association with heat tolerance and tropical adaptation (Mariasegaram et al., 2007; Dikmen et al., 2014; Littlejohn et al., 2014; Porto-Neto et al., 2018). As with many countries, a concern here is public and government acceptance of the new products.

As also described significant efforts are ongoing aimed at discovering genetic variants of economic and ecological significance, primarily using a signature of selection approach. Given the current emphasis on incorporating traits conferring adaptation to harsh (including hotter) environments into breeding programs, both within developed and developing countries, this body of work may gain momentum. In one of the case studies presented the signature of selection study utilized data availed from an African breeding program, which adds value to the performance data collected. Whilst GWAS studies are currently few, additional studies using this approach are also expected as data-sets build up, such as what will be available via the African Dairy Genetics Gain project. Besides feeding into the discovery of genomic variants, GWAS studies can provide useful QTL information for use in genetic improvement programs. The evolutionary history of Africa indigenous livestock species, make African populations a particularly powerful resource for gene discovery (for example, Mwai et al., 2015; Kim et al., 2017), and if genes of significant effects are discovered they could be highly valuable. However, moving from initial results to confirmation of associations and then on to gene discovery requires substantial resources and time. Substantial investment will be required to move from genetic associations to applications in African livestock.

An important issue related to the use of African animal genetics resources is the fair and equitable sharing of benefits arising from their utilization. The Nagoya Protocol on Access to Genetic Resources and the Fair and Equitable Sharing of Benefits Arising from their Utilization to the Convention on Biological Diversity (the Nagoya Protocol^1^) is the critical guiding document to this end. This protocol is a 2010 supplementary agreement to the 1992 Convention on Biological Diversity, which entered into force October 2014. The protocol defines obligations of the providers and users of the all genetic resources in relation to access, benefit sharing and monitoring the compliance of users with legal ABS requirements of the provider country. By default and due to the lack of any specialized international instrument, access to animal genetic resources for food and agriculture for R&D activities would fall under national ABS regulation, if the country did not determine this otherwise. According to information provided in the Access and Benefit Sharing (ABS) Clearing House, a web platform aimed at supporting implementation of the Nagoya Protocol^2^ 43 African countries are currently parties the Nagoya Protocol, though many of these are still developing the related policies and laws as well as implementing procedures and practices. The implementation of the Nagoya Protocol provides both opportunities and challenges for African countries, as discussed in AU-IBAR (2016). To help capitalize on these opportunities, whilst reducing the challenges, additional capacity building of both non-African as well as African actors on the Nagoya Protocol and the implementing legal framework in its Member States is urgently required.

### Future Outlook

Thinking toward the future, for African livestock systems to better capitalize on the potential of livestock genomics several key issues needs to be addressed. Critically these include: establishment of sustainable genetic improvement strategies into which the use of genomic technologies can be embedded; enhanced phenomic capabilities; new genomic tools and/or algorithms designed for application in African livestock population structures; and enhanced capacity in animal breeding, genomics, and genetics. These are discussed in more detail in the following paragraphs.

Few genetic improvement strategies, i.e., breeding programs linked to multiplication and delivery systems that have the potential to produce impact at scale, exist in Africa, with the notable exception of South Africa (which has a highly developed economy and livestock infrastructure, as well as high capacity in animal breeding). For the majority of countries, significant further investment in identifying and establishing context-specific and sustainable genetic improvement strategies are required, which genomic technologies can help enable or into which the genomic application can be embedded. Excellent guidelines to this end are given in FAO (2010), and other useful experiences have also been shared (Kosgey et al., 2011; Philipsson et al., 2011; Haile et al., 2013, 2016; Mueller et al., 2015; Bruno et al., 2016; Mrode et al., 2016; Ojango et al., 2016). Some elements promoted as being key to the success and sustainability of a genetic improvement strategies within Africa are: supportive policy and institutional arrangements; close engagement with all stakeholders to ensure their needs and preferences are met, including in the design stage; incorporation of the private sector; providing incentives for farmer participation, such as timely feedback on their own animals for enhanced farm-management decision making; ensuring equality of access to the breeding technologies and information, including from a gender perspective; and awareness raising of livestock keepers and other stakeholders on the value of genetic improvement, particularly when packaged with other interventions, such as animal health-care and feeding, that allow the improved genetics to be expressed.

In initiatives where both phenotypes and genotypes are required, the phenotypic information is usually more expensive and difficult to obtain than the genotypic information, particularly as the cost of genotyping declines (Biscarini et al., 2015). To this end phenotyping tools that are cheap, reliable and easy to use are required. Once such example is the use of weigh-bands (tape measures placed around the girth of an animal from which the animal’s weight can be read) in cases where farmers do not have access to weighing scales (for example, Tebug et al., 2016). Whilst many other ‘higher tech’ examples exist, such as wearable devices for remote recording of livestock health, movement and reproductive status (Rutten et al., 2013; Egger-Danner et al., 2015), and several are being tested in African systems, these are currently not affordable by the majority of livestock keepers in Africa.

Phenomic tools extend beyond recording into methods of analysis. Production systems, population structures and data quality of many African livestock populations differ markedly from the intensive systems in which most existing phenotype and genetic data analysis methods have been developed and tested. It can be expected that this will, at the very least, often lead to very different phenotype and genetic parameter values than typically seen in intensive systems. In many cases, statistical models will need to be developed that are appropriate for the population. For example, for smallholder dairy systems, typical herd-year-season effects cannot be applied (because of the very small number of cows per herd), methods of fitting lactation curves may not be appropriate to lactations that do not exhibit a classical lactation curve and/or the shape of the curve is highly dependent on production level, and variation across the lactation may be high due to short-term fluctuations in feed availability. Additionally, factors such as genotype by environment (GxE) interaction that typically have modest effects in intensive systems, where environmental differences between farms are typically relatively small, may be much more imported in African livestock systems. For example, smallholder crossbred dairy farms in East Africa range from under 1,000 l milk/cow/annum to over 5,000 l milk/cow/annum. There is massive GxE in terms of breed composition (high grade or pure exotics do best in the best environments while low-grade exotics perform best in the poorest environments) and hence it should be expected there will be large GxE when undertaking genetic evaluations in such populations. It will be important to ensure that existing methods of analysis drawn from the global literature are properly tested and adapted where needed to provide appropriate analyses for African livestock populations.

African livestock genetic research, development and application has a huge advantage in being able to utilize the wide range of genomic tools that have been developed for use globally. Most notably the existing genome sequence assemblies and associated annotations coupled with the various commercially available SNP genotyping assays provide immediate tools for analyses of genetic diversity, genetic evaluations, signatures of selection, GWAS and gene discovery. However, all of these tools were developed with little or no information from African livestock populations. It is not yet known whether updated or customized assays will be required to obtain the maximum utility in African populations, though in the case of cattle work is being undertaken to this end (ILRI, 2016). As a precursor to the work on imputation of SNP data in East African crossbred cattle populations (Aliloo et al., 2018) it was shown that the bovine high density assay with 777,000 SNP was highly informative for African indigenous cattle populations, in the sense that it has more than 190,000 markers with high minor allele frequency for most cattle populations tested. However, it also showed that the existing commercial 7 k SNP assay had low power for imputation in crossbred populations (Aliloo et al., 2018). Related to this, imputation algorithms will need to be developed for African pure and crossbred populations, as Aliloo et al. (2018) did for the East African crossbred dairy cattle. The degree of shared LD between African indigenous populations is not yet known but, as is the case for developed world breeds, it is not expected to be high. So imputation algorithms will need to be trained for each population separately or trained on a population of animals sampled from a variety of breeds, as has worked well for some minor breeds in developed countries. Although the existing high density (>600 k) SNP assays are expected to work well for basic GWAS in all populations, they may remain suboptimal on two levels: (a) we are lacking the sequence information to impute up to full sequence data for African populations plus the assays may not have an ideal SNP set to allow imputation to sequence variants that exist in African populations; (b) the information content of the existing SNP may not allow accurate separation of the indigenous versus exotic versus between-breed LD and hence not allow an advanced (and hence accurate) GWAS to be performed in crossbred populations. As more information accumulates it will be become clear how much value improved assays will add for each of the livestock species in Africa. Given that current genotyping platforms have a strong negative relationship between volume of sales and price, this value can be assessed against the cost relationship to determine the cost-benefits of developing customized assays for each species.

Building human capacity in animal breeding, genetics and genomics within Africa, such that appropriate expertise exists to design and support implementation of the genetic improvement strategies and linked genomic technologies, is required. Suggestions on how to strengthen developing country higher education systems in animal breeding are given by Ojango et al. (2008). These include concerted efforts in training of trainers, co-operation among higher education institutes within regions (South–South collaboration) in order to improve the quality of training offered, and collaboration with institutes in more developed countries. A formal on-line discussion forum revealed that the needs for human capacity development in livestock genetics and breeding go far beyond expanding post-graduate training (Chagunda et al., 2015). Principal among the needs was the current lack of effective career and mentoring structures for post-graduates trained in livestock genetics and breeding, such that most such graduates end up working in other disciplines or lacked support to evolve from a trained post-graduate to become and expert practitioner. Sharing of learning lessons across genetic improvement initiatives within Africa would also be extremely valuable, and additional efforts to this end are warranted.

### Concluding Comments

In conclusion, genomic applications are currently benefiting African livestock systems in a variety of ways, including on genetic improvement and more broadly, such as assisting in system characterization. This has emerged relatively recently, largely within the last 5 years. The expectation for the future is that African livestock systems will increasingly benefit from genomics, particularly if the various issues constraining this (as discussed in this paper) are addressed. The rate at which this will occur will large depend on the level of investment in African livestock genetic improvement.

## Author Contributions

KM and JG were the main authors of the manuscript, with information on the case studies supplied by JG, OM, RM: Kenya dairy cattle and East Africa dairy cattle, KM: Senegal dairy cattle, TG: Ethiopia sheep, SK: trypanosome resistant cattle, AH: Ethiopia small ruminants, and JM: other initiatives on genomic variant discovery.

## Conflict of Interest Statement

The authors declare that the research was conducted in the absence of any commercial or financial relationships that could be construed as a potential conflict of interest. The reviewer GM declared a past co-authorship with several of the authors TG, AH, and OM to the handling Editor.

## Footnotes

1 https://www.cbd.int/abs/text/default.shtml

2 https://absch.cbd.int/
